# Evaluation of the effect of longitudinal connectivity in population genetic structure of endangered golden mahseer, *Tor putitora* (Cyprinidae), in Himalayan rivers: Implications for its conservation

**DOI:** 10.1371/journal.pone.0234377

**Published:** 2020-06-15

**Authors:** Prabhaker Yadav, Ajit Kumar, Syed Ainul Hussain, Sandeep Kumar Gupta

**Affiliations:** 1 Department of Animal Ecology and Conservation Biology, Wildlife Institute of India, Dehradun, India; 2 Department of Landscape Level Planning and Management, Wildlife Institute of India, Dehradun, India; SOUTHWEST UNIVERSITY, CHINA

## Abstract

In many aquatic species, alteration of habitats and human-induced barriers shape the population’s genetic structure in rivers with longitudinal connectivity. The golden mahseer, *Tor putitora* (GM) is an endangered and sensitive cyprinid species. It is considered an indicator of a healthy freshwater ecosystem and is found in cold-water habitats. Therefore, it is crucial to understand how longitudinal connectivity and anthropogenic factors affect the diversity and population genetic structure of GM. The population genetic structure, gene flow and demography of the GM in four Himalayan rivers were investigated by mitochondrial cytochrome *b* gene (cyt *b*) as well as microsatellite genotyping. The results showed overall high mtDNA diversity (hd: 0.795) couple with low nucleotide diversity (π: 0.0012) in all GM populations. We also found significant levels of observed heterozygosity (ranging from 0.618 to 0.676), with three genetic clusters. The mtDNA and microsatellite analysis suggested that there are close genetic relationships between the Bhagirathi and Ganga populations; whereas, significant level of genetic differentiation was observed with that of Alaknanda and Yamuna populations. Haplotype distribution, unimodal distribution graph and results of the neutrality test indicated a sign of recent population growth in the GM population. Analysis of molecular variance (AMOVA) and spatial molecular variance (SAMOVA) revealed existence of genetic structures in GM populations. In addition, spatial genetic analysis detected a significant correlation between the pairwise genetic and geographical distances for the entire study area (Mantel test, *rM* = 0.126; *P* = 0.010). Considering the significant level of heterozygosity, high rate of unidirectional migration and the intra-population structuring in Alaknanda and Yamuna, it is crucial to propose an effective conservation plan for the GM populations. In general, dams obstruct continuous water flow and create isolated microhabitats. Therefore, we recommend the establishment of microscale protected areas near GM breeding sites and construction of fish pass to maintain the genetic connectivity of fish species that enhance viable populations.

## Introduction

The Himalayan riverine region has a rich diversity of species and unique bio-geographic features. The Ganga and Yamuna, the major Himalayan rivers, have been designated a lifeline of India. They flow from the Western Himalaya and converge at Triveni Sangam, Prayagraj, Uttar Pradesh and drain into the Bay of Bengal. Hydro-power development and construction of dams and barrages in the Himalaya have significantly affected the natural habitat, abundance, and population structure of aquatic animal species over the last 45–50 years [1–4]. The barriers affect the movements of these species, creating separate sub-populations. When a species has adapted to a particular habitat, it often becomes restricted to a small patchy habitat, with limited gene flow [4]. Understanding the genetic structure in continuous habitats is essential for determining the effects of these barriers on the genetic diversity and gene flow in populations and developing appropriate conservation programs [5].

**Fig 1:**
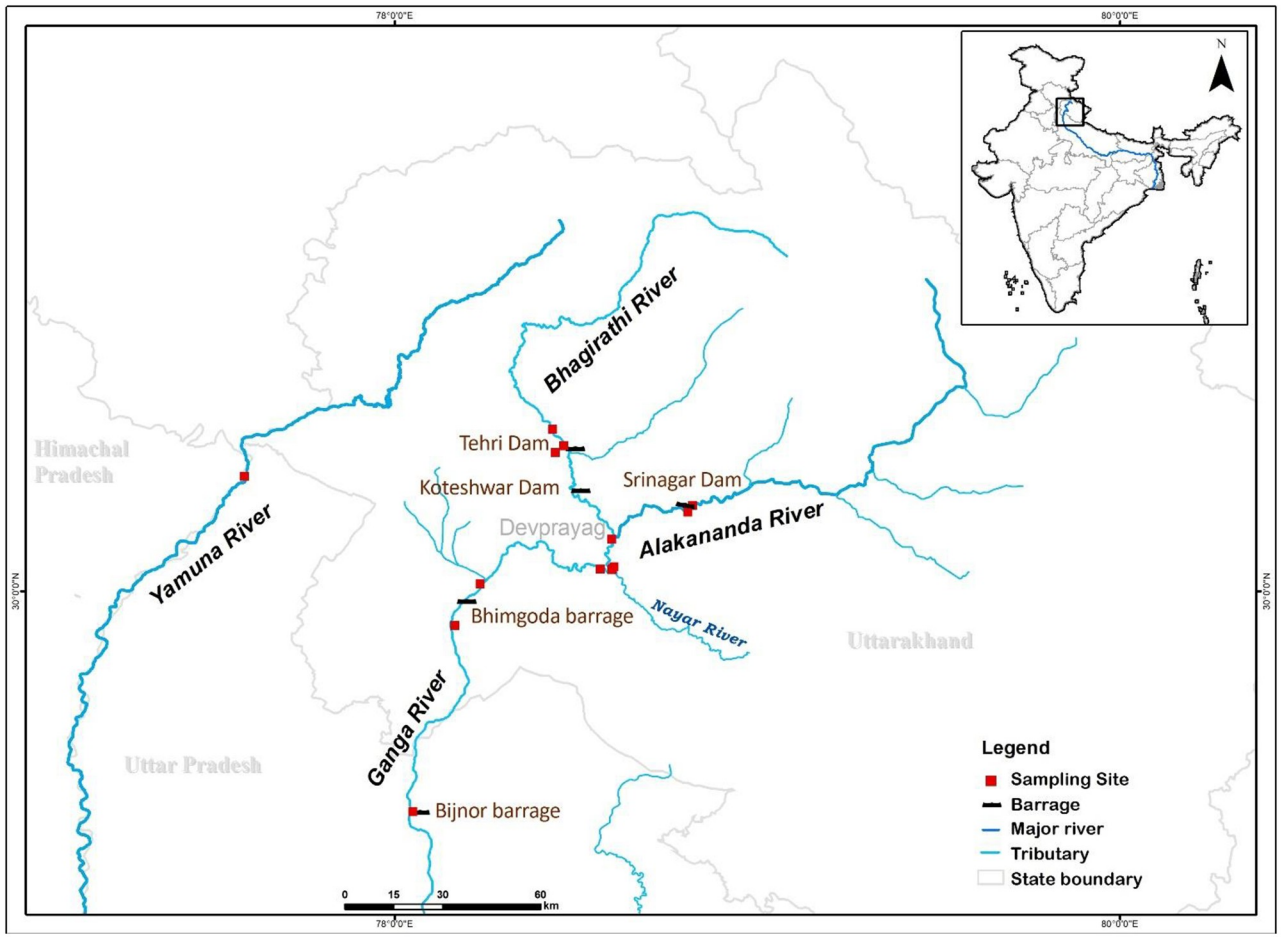
Map of study area showing the golden mahseer population sampling sites.

The golden mahseer (*Tor putitora*, GM), is a flagship species, belongs to the family Cyprinidae [3]. It is an important cold-water fish species where the temperature ranges between 11°C to 24°C, while temperature for spawning grounds have been reported in the range of 11°C to 30.5°C in the foothills of the Himalayan region [6–8]. Due to its large body size (growing up to 54 kg) and striking golden color, it is a preferred sport fish [2,9]. The GM is a rheophilic and sensitive species. Its presence is an indicator of a healthy freshwater ecosystem [9,10]. The natural range of the GM is declining due to reduced water levels/flows, increasing river water temperatures and overexploitation [8,11,12]. Thus, it is listed as Endangered in the IUCN Red List [13]. The distribution range and population biology have been done from Indian rivers, but detail population genetic studies of GM is still lacking [1,2,8,14–16]. Despite this, no genetic assessment has been carried out in the Ganga and Yamuna river; however, a few genetic studies have been conducted in other rivers [17–22]. Barriers created by humans may have the potential to cause genetic differentiation and structuring in populations that were previously continuous [23,24]. Little is known about the effect of dams on the gene flow of GM in continuous habitats, specifically the tributaries of river Ganga (Bhagirathi and Alaknanda, which have Y-shaped connectivity and Yamuna river) (Fig 1). In this study, we used variations of the mtDNA Cyt *b* gene and microsatellite markers to describe the population genetic structure, gene flow, and demography of the GM in the Bhagirathi, Alaknanda, Ganga, and Yamuna rivers. We have also attempted to address the impact of dams and barrages on the genetic structuring of the GM.

## Results

### mtDNA Cytochrome *b* sequence polymorphism and genetic diversity

We obtained the complete mtDNA cyt *b* region of 1140 bp from 201 GM (Bhagirathi = 78, Alaknanda = 26, Ganga = 42, and Yamuna = 55). After sequence alignment, we obtained 27 variable sites among the populations, accounting for 2.36% of the entire number of sites. Of these, 14 sites were singletons, and 13 were parsimony informative. The estimated transition/transversion bias (*R*) was 1.55. The typical nucleotide composition was T = 27.7%, C = 28.3%, A = 30%, and G = 14%; these values indicate A+T rich region among the mitochondrial cyt *b* gene.

**Fig 2:**
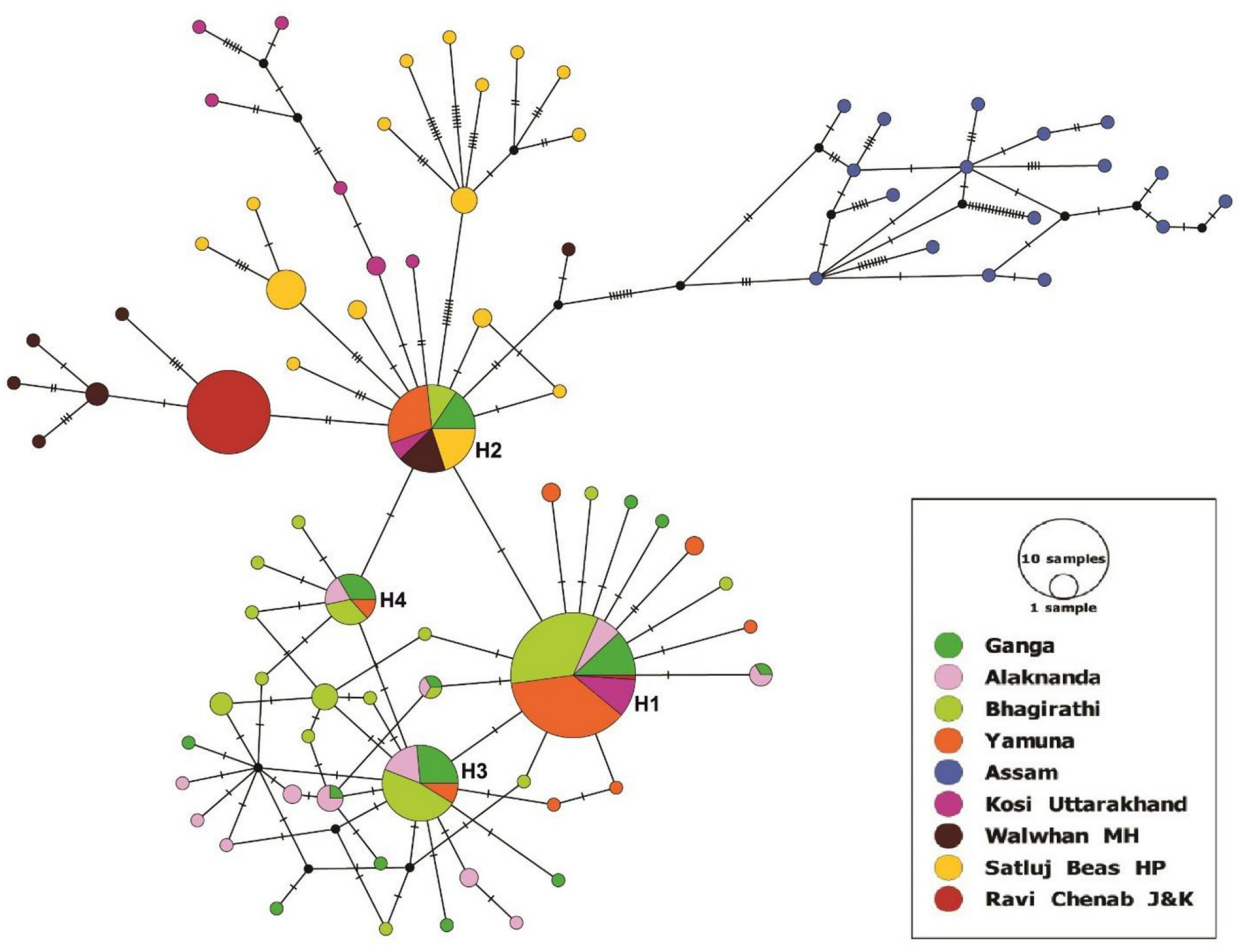
A median-joining network of golden mahseer haplotypes. The major haplotypes (H1-H4) were labeled. The size of each circle indicates the relative frequency of the corresponding haplotype in the whole dataset.

The sequences were grouped into 38 haplotypes (hap) (Table 1). The haplotypes of GM were deposited in GenBank with the accession number: MK783224–MK783261. We detected 17 haplotypes (hap1–4, hap21–33) in the Bhagirathi, 11 haplotypes (hap1, hap3, hap4, hap7, hap12, hap15–20) in Alaknanda, 14 haplotypes (hap1–14) in the Ganga, and nine haplotypes (hap1–4, hap34–38) in the Yamuna. Three haplotypes (hap1, hap3, hap4) were shared between the populations of the Bhagirathi, Alaknanda, Ganga, and Yamuna. Majority of individuals 81, 25, 34 and 15 clustered in the hap1, hap2, hap3 and hap4, respectively. Many private haplotypes were also identified in all the populations (Fig 2). The haplotype diversity values (hd) of the Bhagirathi, Alaknanda, and Ganga were almost equal and high (>0.78). In contrast, the haplotype diversity of the Yamuna population was comparatively low (0.62) (Table 2). Overall, the haplotype diversity was high and the nucleotide diversity was low in the examined GM populations (hd, 0.795; π, 0.00127).

**Table 1:** Haplotype distribution of Cytochrome *b* gene in four populations of the golden mahseer.

Haplotype	Bhagirathi	Ganga	Alaknanda	Yamuna	Accession numbers
Hap_01	32	11	6	30	MK783224
Hap_02	6	7		13	MK783225
Hap_03	16	9	6	3	MK783226
Hap_04	5	5	3	2	MK783227
Hap_05		1			MK783228
Hap_06		1			MK783229
Hap_07		1	2		MK783230
Hap_08		1			MK783231
Hap_09		1			MK783232
Hap_10		1			MK783233
Hap_11		1			MK783234
Hap_12	1	1	1		MK783235
Hap_13		1			MK783236
Hap_14		1			MK783237
Hap_15			1		MK783238
Hap_16			1		MK783239
Hap_17			1		MK783240
Hap_18			2		MK783241
Hap_19			1		MK783242
Hap_20			2		MK783243
Hap_21	1				MK783244
Hap_22	1				MK783245
Hap_23	1				MK783246
Hap_24	1				MK783247
Hap_25	4				MK783248
Hap_26	3				MK783249
Hap_27	1				MK783250
Hap_28	1				MK783251
Hap_29	1				MK783252
Hap_30	1				MK783253
Hap_31	1				MK783254
Hap_32	1				MK783255
Hap_33	1				MK783256
Hap_34				1	MK783257
Hap_35				1	MK783258
Hap_36				2	MK783259
Hap_37				2	MK783260
Hap_38				1	MK783261

**Table 2:** Sample size (N), number of haplotypes (H), haplotype diversity (Hd), nucleotide diversity (π), Fu’s F*S*, Tajima’s D, mismatch distribution test (SSD) and raggedness index (r) in four populations of golden mahseer (*Tor putitora*). *P* is the probability value.

Populations	N	H	Hd	π	Fu’s F*S* (*P*)	Tajima’s D (*P*)	SSD (*P*)	r (*P*)
Ganga	42	14	0.878	0.00151	-8.493 (0.000)	-1.318 (0.081)	0.0064 (0.180)	0.0915 (0.050)
Bhagirathi	78	18	0.785	0.00123	-13.083 (0.000)	-1.0239 (0.156)	0.0015 (0.510)	0.0608 (0.290)
Alaknanda	26	11	0.889	0.00151	-5.457 (0.001)	-0.777 (0.234)	0.0128 (0.130)	0.0886 (0.170)
Yamuna	55	9	0.629	0.00078	-4.470 (0.007)	-1.067 (0.147)	0.0035 (0.320)	0.0849 (0.200)
Total	201	38	0.795	0.00127	-7.876 (0.002)	-1.046 (0.154)	0.0061 (0.285)	0.0815 (0.177)

### mtDNA structure and network analysis

Sequences of GM from seven other different Indian rivers, namely the Ravi (Basoli, Jammu & Kashmir, n = 22), Beas (Joginder Nagar, Himachal Pradesh, n = 16), Satluj (Bhakara, Himachal Pradesh, n = 21), Chenab (Anji, Jammu & Kashmir, n = 21), Kosi (Ram Nagar, Uttarakhand, n = 19), Jia Bhoreli (Bhalukpong, Assam, n = 17), and Walwan dam on the Indrayani (Lonavala, Maharashtra, n = 16) were taken from GenBank to determine the inclusive mtDNA structure and to carry out network analysis [22]. We detected 83 haplotypes from 333 GM sequences. Of these, 47 haplotypes had been reported by Sati et al. (2015) [22] from seven rivers, and only two haplotypes (H1 and H2) of her study were shared with our data (Fig 2).

The median-joining (MJ) network of the 83 haplotypes represented the distribution pattern of haplotypes among the GM populations (Fig 2). The haplotypes genealogy showed four core sharing haplotypes (H1-H4), whereas the majority of the haplotypes contained a single individual. The sequences of Jia Bhoreli (Assam) did not share haplotypes with the other GM populations and formed a distinct cluster. Sharing of haplotypes was more common in the Bhagirathi, Alaknanda, Ganga, and Yamuna. The haplotype H1 was grouped with the sequence of the Kosi and Ravi-Chenab, whereas H2 was shared with Kosi, Indrayani (Walwhan, MH), and Satluj-Beas. We also attempted to construct the phylogenetic relationships among all the GM populations; however, due to low intraspecific sequence divergence, the clade of different populations could not be resolved except Jia Bhoreli river, Assam (S1 Fig).

### Demographic history

**Fig 3:**
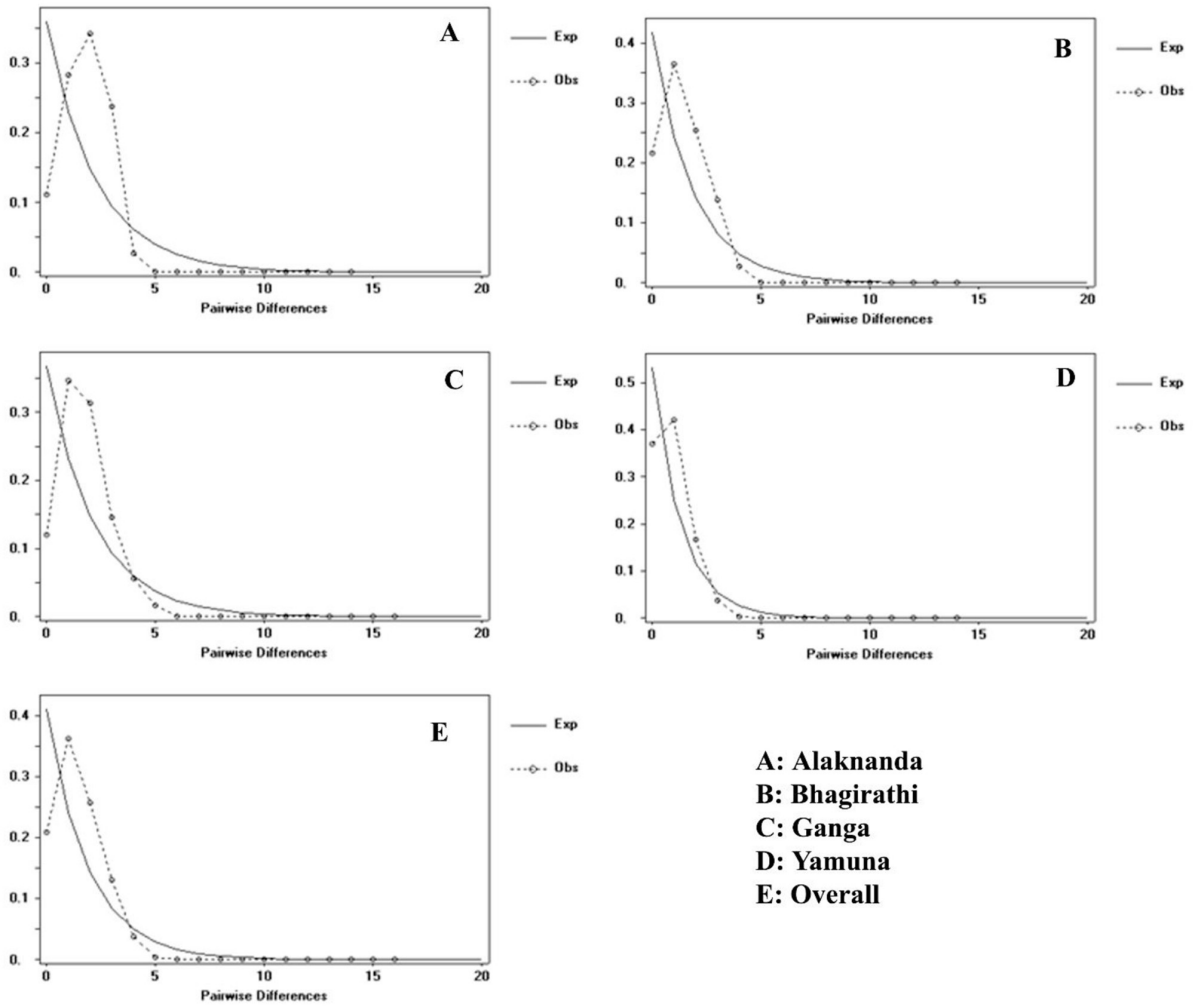
Mismatch distribution graphs for golden mahseer populations. The X and Y axis show the number of pairwise differences and the frequency of the pairwise comparisons, respectively. The observed frequencies are represented by dotted line. The frequency expected under the hypothesis of constant population model is depicted by solid line.

The neutrality test of Tajima’s D and Fu’s Fs tests were carried out to deduce the demographic history of the GM (Table 2). Negative neutrality test values, indicating an excess of rare nucleotide site variants under a neutral model of evolution. Here, we observed negative D but statistically not significant for all GM populations. The large significant negative Fu’s Fs values were observed in all GM populations, which showed that there is an excess of rare mutations and will be taken as evidence of deviation caused by population growth or selection. A historical demographic expansion model was used to obtain the frequency of pairwise distribution among sequences. Unimodal plots of the mismatch distribution were observed in the studied GM populations (Fig 3), indicating the existence of different groups of haplotypes that corresponded to the populations. The demographic scenario was also supported by the generalized least square procedure (SSD = 0.0061, *P* = 0.285) and the raggedness index of the distribution (Rag = 0.0815, *P* = 0.177) (Table 2).

### Microsatellite analysis

#### Genetic variations and diversity in four riverine populations

Out of 11 microsatellites, two loci (TPM04 and TPM15B) showed very less heterozygosity; hence those were excluded from analysis; therefore, nine loci were used to calculate the genetic diversity of the GM populations of the four rivers. The comparison of allele size for each markers obtained in the present study to the original source has been provided in the S1 Table. The PIC values of microsatellite ranged from 0.316 (BARB37) to 0.870 (BARB59), with an average of 0.656. The number of alleles per locus varied from five (BARB37) to 17 (TPM01), with a mean of 11.2. The mean number of effective alleles (Ne) was ranged from 8.43(BARB59) to 1.609 (BARB39), with an average of 3.891. The numbers of alleles per locus and PIC value (>0.5) indicated that the loci used in our study were very polymorphic. No linkage disequilibrium was detected (P > 0.05). The mean observed and expected heterozygosity values were 0.673 and 0.691, respectively. The observed heterozygosity (*H*_*O*_) was comparable (0.67) in the Ganga, Bhagirathi and Yamuna populations, whereas it was low in Alaknanda (0.618). However, the expected heterozygosity (*H*_*E*_) was highest in Ganga and Bhagirathi (0.692), followed by the Alaknanda (0.668) and Yamuna (0.626). The estimated inbreeding coefficient (*F*_*IS*_) value of the populations ranged from -0.070 in the Yamuna to 0.095 in Alaknanda, and the overall value was 0.028 (Table 3). The negative *F*_*IS*_ value, indicating heterozygosity excess in the Yamuna population (Table 3).

**Table 3:** Genetic diversity inferred from nine microsatellite markers for the golden mahseer populations of four rivers.

River		MFW11	MFW17	MFW26	BARB37	BARB59	TPM01	TPM11	TPM18B	TPM21A	Across loci
**Ganga**	Na	9	12	9	3	9	13	6	9	7	8.5
(n = 42)	Ne	2.982	3.564	2.769	1.827	7.320	3.637	3.862	4.552	2.838	3.705
	Ho	0.600	0.667	0.683	0.513	0.786	0.619	0.714	0.833	0.667	0.675
	He	0.664	0.719	0.639	0.453	0.863	0.725	0.739	0.784	0.648	0.692
	P	0.000	0.000	0.009	0.759	0.586	0.081	0.789	0.212	0.000	
	FIS	0.109	0.085	-0.057	-0.120	0.102	0.158	0.045	-0.056	-0.017	0.036
**Bhagirathi**	Na	7	13	11	2	11	11	6	8	6	8.3
(n = 78)	Ne	2.905	2.688	3.774	1.600	7.650	4.482	4.157	4.266	3.019	3.838
	Ho	0.628	0.610	0.803	0.375	0.779	0.756	0.821	0.654	0.654	0.675
	He	0.656	0.628	0.735	0.377	0.869	0.777	0.759	0.766	0.669	0.692
	P	0.000	0.779	0.000	0.003	0.220	0.000	0.125	0.509	0.357	
	FIS	0.048	0.035	-0.085	-0.328	0.110	0.033	-0.074	0.152	0.029	0.011
**Alaknanda**	Na	5	8	8	3	7	11	5	8	3	6.4
(n = 26)	Ne	2.463	3.038	2.948	1.596	5.631	5.026	3.355	4.744	2.537	3.482
	Ho	0.654	0.577	0.640	0.478	0.640	0.885	0.615	0.769	0.308	0.618
	He	0.594	0.671	0.661	0.373	0.822	0.801	0.702	0.789	0.606	0.668
	P	0.910	0.596	0.229	0.518	0.038	0.001	0.091	0.257	0.008	
	FIS	-0.081	0.159	0.052	-0.260	0.241	-0.085	0.143	0.045	0.507	0.095
**Yamuna**	Na	5	8	7	3	11	9	7	6	8	7.1
(n = 55)	Ne	1.452	4.192	2.862	1.450	6.402	2.679	3.858	4.228	2.694	3.313
	Ho	0.309	0.722	0.811	0.370	0.836	0.673	0.782	0.909	0.673	0.676
	He	0.311	0.761	0.651	0.311	0.844	0.627	0.741	0.763	0.629	0.626
	P	0.000	0.258	0.005	0.425	0.008	0.748	0.427	0.383	0.000	
	FIS	0.016	0.061	-0.238	-0.184	0.018	-0.064	-0.046	-0.182	-0.061	-0.070
**All Populations**	Na	10	16	14	5	12	17	9	10	8	11.2
(n = 201)	Ne	2.496	3.635	3.216	1.609	8.432	3.960	4.132	4.608	2.932	3.891
	Ho	0.538	0.648	0.759	0.464	0.779	0.721	0.761	0.776	0.617	0.673
	He	0.599	0.724	0.689	0.379	0.881	0.747	0.758	0.783	0.659	0.691
	FIS	0.105	0.108	-0.099	-0.223	0.119	0.037	-0.002	0.011	0.066	0.028
	PIC	0.559	0.708	0.665	0.316	0.870	0.716	0.720	0.749	0.602	0.656

*Na* number of different alleles, *Ne* Number of effective alleles, *Ho* observed, heterozygosity, *He* expected heterozygosity, *FIS*, Inbreeding coefficient, *PIC* Polymorphic information content, *P* probability of Hardy-Weinberg Equilibrium (*HWE*). Significant values P<0.05.

#### Population structure and genetic differentiation

**Fig 4:**
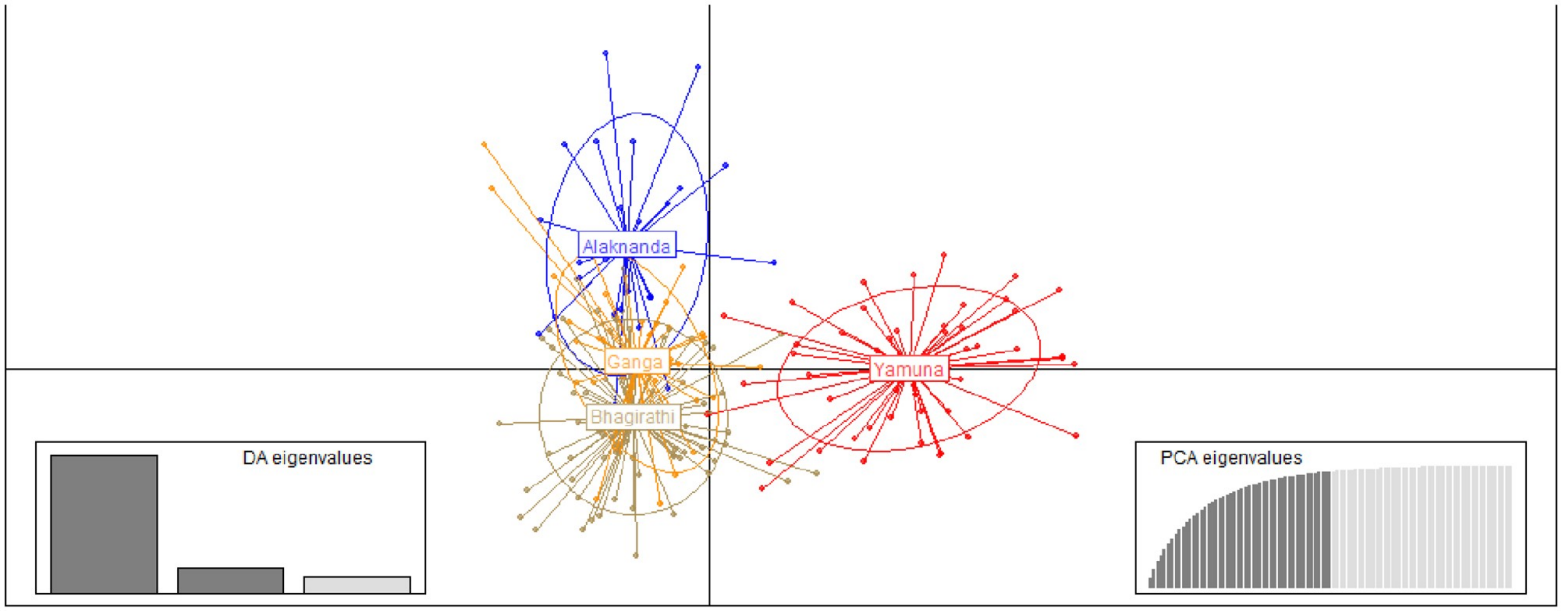
Scatterplots of DAPC based on microsatellite loci for four golden mahseer populations. DAPC carried out using hierarchical islands model and shown by different colors and inertia ellipses. Dots represent individuals. The DA and PCA eigenvalues of the analysis are displayed in inset.

**Fig 5:**
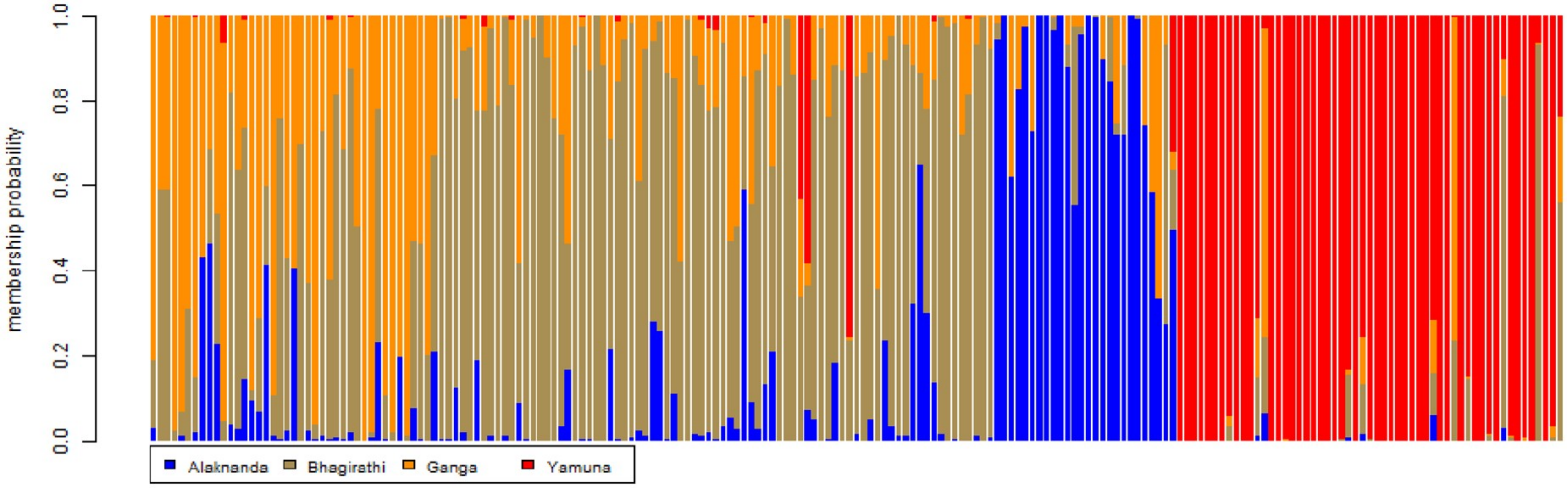
Results of bar plot implemented in Discriminant Analysis of Principal Components (DAPC) based on microsatellite data. Each column along the X axis represents one golden mahseer individual. The Y axis represents the assignment of membership probability of each individual.

We used DAPC to analyze the genetic structure of the GM population. We found that there are three genetic clusters. Cluster 1 consisted of the populations of the Ganga and Bhagirathi, and Cluster 2 and Cluster 3 were the populations of the Alaknanda and Yamuna, respectively. The scatterplots and stacked bar graphs of the assignment probabilities of GM individuals indicated that the Yamuna population was significantly differentiated from the Bhagirathi, Alaknanda, and Ganga populations. Additionally, the Alaknanda’s GM exhibited a distinct but closer genetic relationship with the GM of the Ganga and Bhagirathi. The assignment probability of the GM indicated that individuals of the Bhagirathi and Ganga have a similar genetic structure and cluster with each other (Figs 4 and 5).

**Fig 6:**
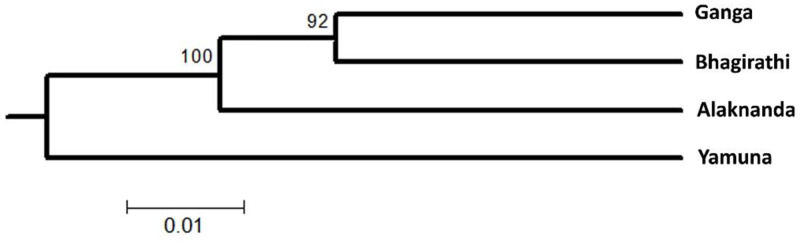
NJ tree of golden mahseer populations from four rivers using microsatellite markers based on Nei’s DA distance with bootstrap values from 1,000 replications.

The level of pairwise genetic differentiation was significantly different from zero in the GM population. The genetic differentiation at Cyt *b* was lower between the Ganga and the Bhagirathi (*F*_*ST*_ = 0.0077) than the Ganga and the Alaknanda (*F*_*ST*_ = 0.0584), whereas it was slightly higher between the Alaknanda and the Bhagirathi (*F*_*ST*_ = 0.0772). A higher genetic distance was observed between the Yamuna to Ganga (*F*_*ST*_ = 0.1008), Alaknanda (0.292), and Bhagirathi (0.109). A similar *F*_*ST*_ pattern was also observed with the microsatellite loci. The low pairwise genetic distance was obtained between Ganga and Bhagirathi (*F*_*ST*_ = 0.007), whereas it was higher between the Alaknanda and Yamuna (*F*_*ST*_ = 0.032) (Table 4). The neighbor-joining dendrogram based on Nei’s DA genetic distance generated from the microsatellite markers indicated that the populations of the Ganga and the Bhagirathi are clustered in one clade, followed by the Alaknanda population whereas the Yamuna GM formed a basal clade (Fig 6).

**Table 4:** Genetic differentiation among the four golden mahseer populations. The pairwise *FST* values based on the mtDNA Cyt *b* gene and microsatellite loci are shown below and above the diagonal, respectively.

	**Ganga**	**Bhagirathi**	**Alaknanda**	**Yamuna**
**Ganga**		0.007	0.012	0.031
**Bhagirathi**	0.0077		0.012	0.029
**Alaknanda**	0.0572	0.0772		0.032
**Yamuna**	0.1008	0.1093	0.2927	

AMOVA indicated that there was phylogeographic structuring in our data. The results of this analysis demonstrated that most of the variances were found within the population of GM (96.28% for the microsatellite markers and 88.31% for the mtDNA Cyt *b* gene) rather than among the populations (0.480% for the microsatellite markers and 1.053% for the mtDNA Cyt *b* gene), suggesting that there is significant genetic structuring within the group. The fixation index among groups (*F*_CT_) except microsatellite, among populations within groups (*F*_SC_) and within populations (*F*_ST_) were statistically significant (P<0.05) (Table 5). The SAMOVA analysis identified maximally differentiated groups in golden mahseer. In the SAMOVA analysis, The number of clusters (K value) ranged between 2 to 8. High genetic differentiation between groups was detected, with *F*_CT_ values ranging from 0.650 to 0.890. The *F*_CT_ values were always statistically significant (except K = 2 and K = 4). *F*_CT_ value was highest at K = 3, (0.806; P<0.05) with *F*sc (0.030, P<0.001) in this case, GM from Jia Bhoreli, Assam and Walwan dam, MH became separated, and leaving other populations. With an increase of K value up to K = 8, Ganga and Bhagirathi GM were still attribute to one group, whereas Alaknanda, Yamuna, and the rest of the population created a separate group (Table 6).

**Table 5:** Analysis of molecular variance (AMOVA) of four populations of golden mahseer.

Source of variation	Microsatellite markers					mtDNA Cyt *b* gene				
	d. f.	Sum of squares	Variance components	% of variation	Fixation index (*P*)	d. f.	Sum of squares	Variance components	% of variation	Fixation index (*P*)
Among groups	2	34.425	0.1119 Va	3.24	*F*CT: 0.032 (0.161)	2	11.288	0.0823 Va	10.78	*F*CT:0.1078 (<0.05)
Among populations within groups	1	5.122	0.0164 Vb	0.480	*F*SC: 0.0049 (P<0.05)	1	1.053	0.0070 Vb	0.91	*F*SC:0.0102 (<0.05)
Within populations	398	1323.831	3.3262 Vc	96.280	*F*ST: 0.0324 (P<0.05)	198	133.466	0.6740 Vc	88.31	*F*ST:0.1169 (<0.05)

**Table 6:** Population groups identified by spatial analysis of molecular variance (SAMOVA) algorithm using the mtDNA Cyt *b* dataset.

K	Population grouping	*F*_*CT*_	F_SC_
**k = 2**	[GA, AK, BH, YN, KS, WH, SB, RC] [AS]	0.890^ns^	0.006***
**k = 3**	[GA, AK, BH, YN, KS, SB, RC] [AS] [WH]	0.806*	0.030***
**k = 4**	[GA, AK, BH, YN, KS, WH] [AS] [SB] [RC]	0.723^ns^	0.043***
**k = 5**	[GA, AK, BH, YN, KS] [AS] [WH] [SB] [RC]	0.717**	0.063***
**k = 6**	[GA, AK, BH, YN] [AS] [KS] [WH] [SB] [RC]	0.698**	0.117***
**k = 7**	[GA, BH, YN] [AK] [AS] [KS] [WH] [SB] [RC]	0.674*	0.312***
**k = 8**	[GA, BH] [AK] [YN] [AS] [KS] [WH] [SB] [RC]	0.650*	0.319***

GA, Ganga; AK, Alaknanda; BH, Bhagirathi; YN, Yamuna; KS, Kosi; AS, Assam; WH, Walwan MH; SB, Satluj Beas HP; RC, Ravi Chenab J&K. Significant values *P<0.05; **P<0.01; ***P<0.001, ns: Not significant.

**Fig 7:**
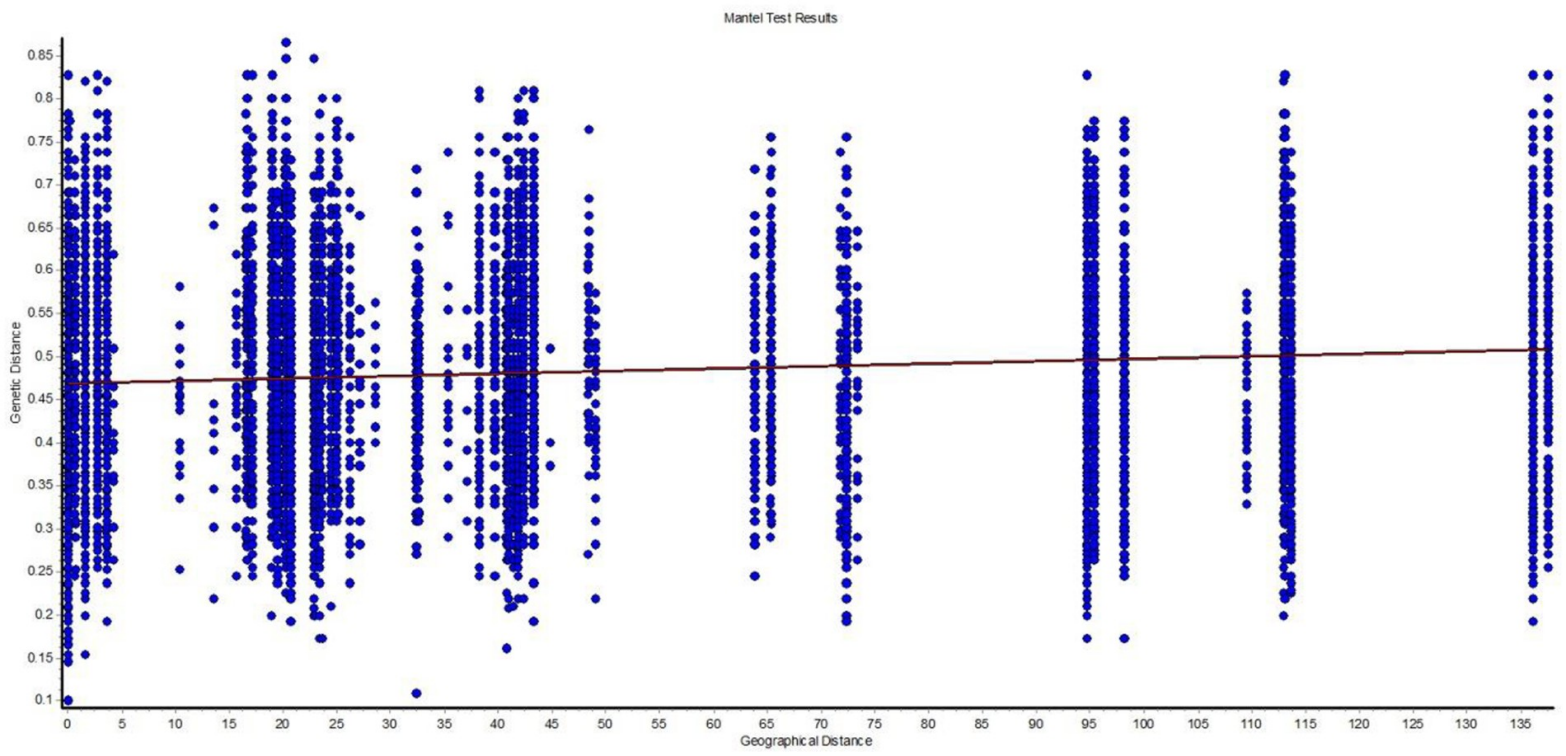
Correlation of genetic distances (F_*ST*_) and geographical distance (km) of golden mahseer samples using microsatellite data. (Mantel test, *rM* = 0.126; *P* = 0.010).

The spatial genetic analysis detected a significant correlation between the genetic and geographical distances for the study area (Mantel test, *rM* = 0.126; *P* = 0.010) (Fig 7). However, this pattern of the isolation by distance (IBD) was strongly influenced by the genetic differentiation (see pairwise *F*_ST_ in Table 4) and the geographical distance between the golden mahseer populations.

#### Population demography and contemporary migration rate

**Fig 8:**
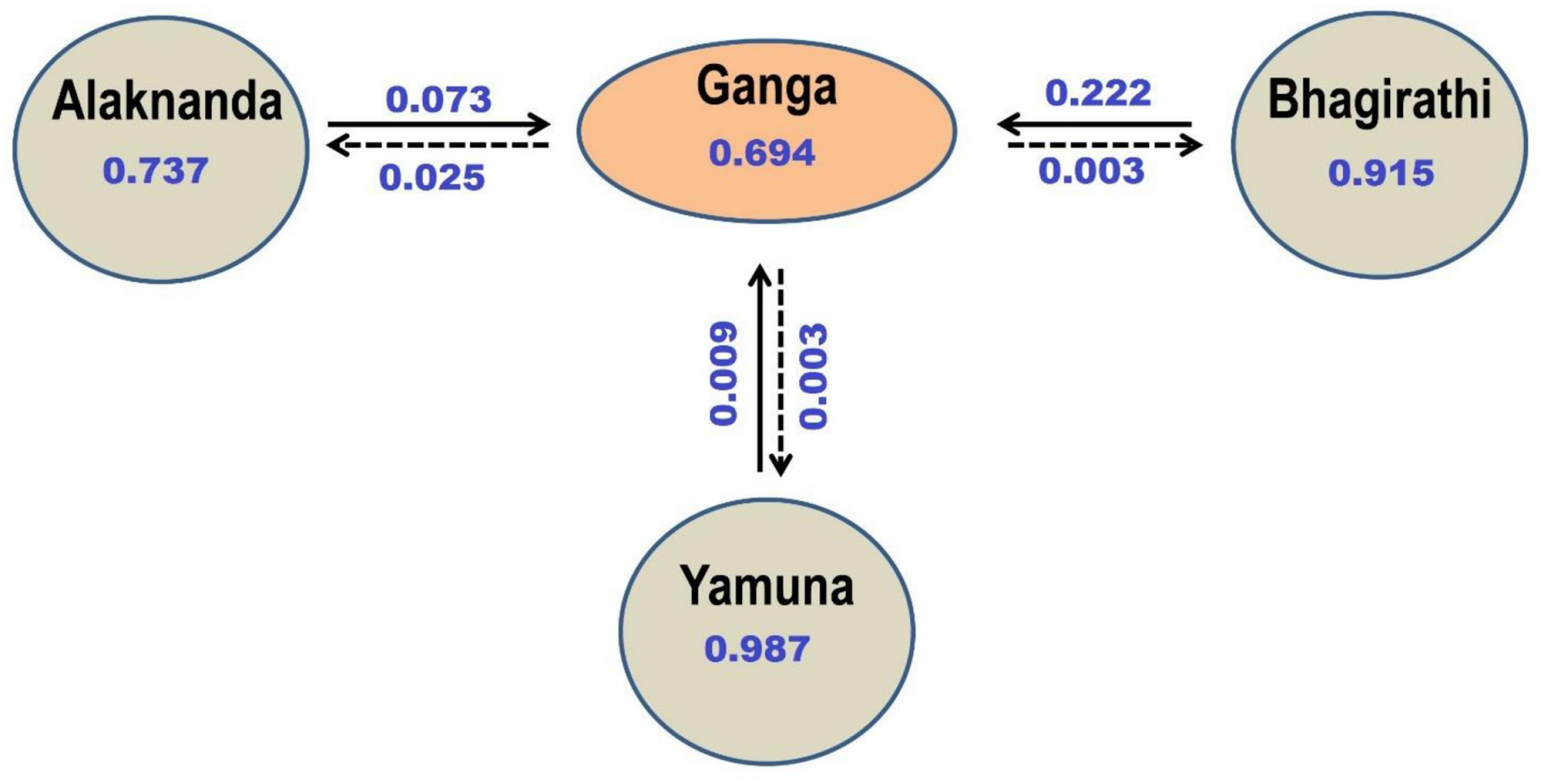
Values of recent migration rates detected with BayesAss program for four golden mahseer population with 95% credible set. Arrows show the direction of gene flow.

The Garza–Williamson index (G-W index; M ratio) test showed values lower than 0.68 (Ganga 0.367; Bhagirathi 0.352; Alaknanda 0.318, Yamuna 0.360), indicated that GM had suffered a historical reduction in population size. Besides, to examine the recent demographic scenario, we analysed the bottleneck test. We did not detect signs of the recent bottleneck in any of the studied GM populations with non-significant values in the Wilcoxon sign-rank test and two mutational models test: Two-phase model (TPM) and step-wise mutation (SMM) that correspond to a one-tailed test of heterozygosity excess. The mode-shift test suggests that there is no distortion of the allelic frequency, and a normal L-shaped distribution was observed in our data set (Table 7). Four independent replications in BayesAss yielded almost similar results. We found high rate of contemporary migration of GM into Ganga from Bhagirathi river (22%), whereas it was very low in the opposite direction (0.3%). However, the migration rate of GM into Ganga from Alaknanda was comparatively low (7.3%), whereas it was also low in opposite direction (2.5%). In addition, a low level of migration was observed from Ganga to the Yamuna and vice versa (Fig 8).

**Table 7:** Results of bottleneck analysis based on microsatellite loci for four populations of golden mahseer.

Population	Mutation model	Wilcoxon test	Mode-shift test	M-ratio (G-W index)
Ganga	T.P.M.	0.993	L-shaped	0.367
	S.M.M.	0.997		
Bhagirathi	T.P.M.	0.912	L-shaped	0.352
	S.M.M.	0.973		
Alaknanda	T.P.M.	0.817	L-shaped	0.318
	S.M.M.	0.973		
Yamuna	T.P.M.	0.938	L-shaped	0.360
	S.M.M.	0.938		

Heterozygosity excess tests were investigated under two-phase model (TPM), and stepwise mutation model (SMM). The P values reported for Wilcoxon signed-rank tests correspond to one-tailed probabilities for heterozygosity excess. The mean *M*-ratio (G-W index) for each population was assessed against a critical value of 0.68.

## Discussion

We have assessed the genetic diversity, population genetic structure, gene flow, and demography of GM based on mtDNA and microsatellite markers. GM is restricted to cold water habitats and is very sensitive to change in water temperature; therefore, small climatic fluctuation may lead to a change in its genetic structure. Construction of dams/barrages and high anthropogenic activities in Himalayan rivers leading to limited dispersal ability, unidirectional migration, and isolation of habitats might have shaped the genetic structure and driven differentiation among the GM populations. The population genetic studies on endangered species are dominant tools for understanding the demographic pattern, which has resulted in the development of conservation and management strategies for species [25, 26].

### Population genetic variation, structure and migration

Our analysis of the variations in the mtDNA Cyt *b* from 201 samples from four major populations of Himalayan rivers exhibited 38 haplotypes. Our data supplement those relating to seven other Indian rivers. In a previous study, 134 GM individuals were sequenced into 47 distinct haplotypes [22]. Strikingly, only two (H1 and H2) of those haplotypes were found in our study. The gene diversity values of the populations of the Alaknanda (h = 0.889), Ganga (h = 0.878), and Bhagirathi (h = 0.785) were comparable with the results reported from the Satluj, Walwah, Koshi, and Beas rivers. However, a slightly lower gene diversity was observed in the Yamuna population (h = 0.629). In contrast with other rivers, no variation in the Cyt *b* gene was reported from the GM samples from the Ravi and Chenab [22]. The high level of haplotype diversity coupled with low nucleotide diversity in our studied GM populations indicated that several mtDNA lineages have evolved in the area by differing in a small number of nucleotides. It is also evident from the haplotype network, which showed a small frequency of single nucleotide variation between haplotypes. The core haplotypes with high genetic relatedness were found in Ganga, Bhagirathi, Alaknanda, and Yamuna. The high frequency of haplotype H1 in the network giving rise to all other haplotypes suggested that this core haplotype could reflect an ancestral lineage that subsequently expanded to the other rivers. The results of our study of the mitochondrial markers provide evidence for a common origin of the GM of the Himalayan rivers.

Furthermore, the microsatellite data indicated the significant level of allelic diversity in all the four populations. The mean numbers of alleles were slightly higher in the Ganga in comparison with the Bhagirathi, Alaknanda, and Yamuna rivers. These results were ecologically supportive, where two rivers Bhagirathi and Alaknanda merge and form the mighty Ganga river. Thus, the admixing of these tributaries populations leads to an increase in the allelic diversity of GM in Ganga. In the Hathini Kund barrage of the Yamuna river, heterozygosity excess was detected. It might be due to the human-mediated non-random mating strategy or presence of small reproductive population size. Another possible explanation could be that we collected samples from a single location, i.e. the upper Hathini Kund barrage of Yamuna; therefore, for better insight, more samples from different locations need to be collected for further study.

We found a high rate of unidirectional migration of GM from the Tehri dam across the Bhagirathi replenishes the population in the Ganga river. It was also confirmed by the scatterplot, DAPC structure, and BayeAss analysis. The DAPC results clearly revealed that most of the genetic characters of the Bhagirathi’s GM were found in the Ganga, whereas clear structuring was observed in the Alaknanda and Yamuna. The BayeAss results indicated a high self-association rate in the Yamuna (98.7%) followed by Bhagirathi (91.5%) and Alaknanda (73.7%), which showed limited migration rates. The proportion of nonmigrants from Ganga was 69%, indicates that the majority of individuals were unimigrated from Bhagirathi and other populations. The result is in concordance with the earlier study on European grayling in a Danish river system, where the main stem population continuously receives individuals and alleles from upstream situated tributaries [24]. Therefore, we also suggest incorporation of GM gene pool from other tributaries with studied rivers that will provide a more robust migration rate among the populations. One of Asia’s highest, Tehri dam, followed by the Koteshwar dam, was constructed on the Bhagirathi river. It has led to a considerable increase in the water level that could serve as a refuge for GM and afford protection from illegal fishing. Also, artificial ranching and continuous harvesting of GM maintain the population size and genetic integrity in the Tehri reservoir. However, the low migration rate of GM from Alaknanda into Ganga and isolation of stocks in the upstream region of the Alaknanda might be due to the development of hydroelectric dam in Srinagar with parallel inside tunnel and channel. It resulted in a low water level in the downstream of the dam that leads to patchy and unfavorable conditions for the GM.

### Genetic differentiation and demography

The genetic differentiation between the Ganga and Bhagirathi was weak, whereas it was comparatively higher in the Alaknanda and Yamuna populations. The pairwise *F*ST value obtained with mtDNA and microsatellite markers indicated that Alaknanda exhibits a significant level of genetic differentiation from Ganga and Bhagirathi populations. It is also supported by microsatellite-based phylogenetic analysis and AMOVA. A significant subdivision among populations was revealed due to variance within populations. The SAMOVA analyses also indicated the existence of genetic structures in GM that have formed in response to barriers to gene flow by fragmentation of habitat. The SAMOVA first unglued the Assam GM, subsequently with increasing numbers of groups, other populations of GM from the highly diverse region were also separated. The fact that the unique haplotypes were also detected in single locality indicates limited gene flow among populations. The limited gene flow in the GM could increase genetic differentiation due to the restricted nature of the habitat. The fine-scale population genetic structuring seems common in a species that is having poor disperse ability [27]; thus, microsatellite-based analysis of IBD using mantel test also showed a significant positive correlation between the pairwise genetic and geographical distances from four studied GM populations i.e. Ganga, Bhagirathi, Alaknanda, and Yamuna. The clear pattern of IBD in the GM population in the current study strongly support by the previous study where limited dispersal capacity plays a vital role in local adaption and dispersal of *S*. *nukiangensis* in continuous water habitat [28].

To check the excess of rare mutations as evidence for population expansion, the test of neutrality Tajima’s D statistic was non-significant, whereas it was significant negative for Fu’s *F*_*S*_ test. Although, analysis of population expansion based on mismatch distribution shows non-significant p-values and unimodal distribution graph, indicates the possibility of allopatric divergence followed by stable population growth in GM. The past demographic fluctuations in population size over time (100 generations) were analysed using the G-W index and bottleneck analysis. The G-W indices of four populations were lower than the critical *Mc* value of 0.68, indicating that GM had experienced a historical reduction in population size. However, it was also suggested that the value of the G-W index was very sensitive for the detection of a population bottleneck because the number of alleles is frequently more reduced than the range by a reduction in population size [29, 30]. Therefore, the G-W statistic is supposed to be very small in the population has been through a bottleneck and close to one in stationary populations. Moreover, new alleles arising from mutations do not essentially increase M-ratios and it changes more slowly than heterozygosity immediately following a population bottleneck [29,30]. It was also supported by bottleneck analysis (TPM, SMM, Wilcoxon sign-rank and Mode shift test), where the analysed microsatellite loci showed an excess of the heterozygosity under the equilibrium (*H*_*EQ*_) within the past 2Ne to 4Ne generations [31]. The mode-shift test showed no distortion of the allelic frequency and a normal L-shape distribution was found in all populations. Hydrological changes and human-induced factors such as artificial ranching and continuous harvesting in upper Tehri dam on Bhagirathi and upper Hathini Kund barrage on the Yamuna could have acted as a factor for the current stable population of GM. Based on the *F*_ST_ values, our findings indicate that Alaknanda’s GM has a significant level of genetic differentiation as compared to the Bhagirathi population. It might be due to tunneling of Alaknanda river water after construction of the Srinagar dam leading to a lowering of water level in the mainstream. Similar results were observed in brown trout, where an increase in genetic differentiation is most likely a result of fragmentation by hydro-power dam [32]. It highlights the need for special conservation efforts on ecological restoration of GM habitat in Alaknanda by maintaining continuity in water level in the mainstream. Moreover, the construction of the fish pass is also required to maintain the genetic connectivity of aquatic species. It would facilitate more individuals to migrate towards Ganga and also in the upward direction, which will ultimately enhance the genetic diversity when migrating GM mates with the Bhagirathi population. The confluence of Nayar and Ganga is a breeding hotspot for GM as it provides a favorable habitat for spawning and maintains food supply for a sustainable population [33,34].

### Implications for management

The GM is a flagship species that is confined to the cold water habitats of Himalayan rivers. Various anthropogenic activities in these sensitive habitats have led to a sharp reduction of the population that was present in the natural environment [11, 35]. Any delays in conservation efforts and implementation of a management decision approach would lead to the extinction of the species. The main purpose of the conservation of an endangered species is to increase the effective population size by maintaining the gene flow and overall genetic diversity. This study detected a structuring pattern in the GM at the microsatellite level, with three genetic groups in the Ganga–Bhagirathi, Alaknanda, and Yamuna. The populations of these rivers must be the focus of a conservation plan to maintain the genetic diversity within the basin. The upper Tehri dam on Bhagirathi and Hathini Kund barrage on Yamuna river, have high water levels and are under surveillance. Therefore, artificial propagation or scientific induced breeding projects are the best solutions to conserve endangered mahseer. Thus, it is necessary to define the Bhagirathi and Ganga River as a refuge that plays a central role in the persistence of a large GM population. Besides, we suggest the establishment of microscale protected areas in Ganga where fishing should be prohibited at the confluence of the Nayar river since it is a GM breeding hotspot. Habitat assessment will be carried out in Alaknanda and Ganga for the enhancement of viable populations and maintaining the gene flow and genetic diversity. Further analysis, with a large sample size from other tributaries, and more genetic markers, may be carried out to assess the putative structuring, genetic diversity and migration pattern of the population for developing an appropriate conservation and management plan. This study will significantly provide insights into the current genetic structure of the GM that would eventually help to develop appropriate strategies for a stock management and conservation program.

## Materials and methods

### Study area

The Alaknanda originates from two glaciers, the Satopanth and Bhagirath Kharak glaciers (elevation 3800 m). The Bhagirathi originates from Gomukh, (elevation 3920 m), the snout of the Gangotri glacier. After flowing for approximately 225 km, these two rivers join at Devprayag and are subsequently known as the Ganga [36]. The Ganga is the largest river in India (the fifth-longest in the world). It has a length of 2525 km [37]. Two major dams, at Tehri and Koteshwar, have been constructed across the Bhagirathi, whereas the Srinagar dam is constructed across the Alaknanda [38–40].

### Sampling and DNA extraction

A total of 201 individuals’ fin and tissue samples were obtained from four populations: Ganga (n = 42), Bhagirathi (n = 78), Alaknanda (n = 26), and Yamuna (n = 55). These samples were collected from local fishermen from river banks being harvested for selling in the fish market, and no animals were captured explicitly for this study. Therefore, Institutional Animal Ethics Permission was not required for this research. All the experiments were carried out in accordance with relevant guidelines and regulations. The samples from the Yamuna river were collected from upstream of Hatnikund barrage and compared with the connected populations of the Bhagirathi–Ganga–Alaknanda river system for their genetic parameters and the level of gene flow. Daily (4:30 to 9:00 am and 5:30 to 8:00 pm) efforts were made to collect biological samples from local fishermen at 13 sites (Fig 1 and S2 Table). The fin and tissue samples collected were preserved in 95% ethanol at room temperature. Total genomic DNA was extracted using the phenol-chloroform method [41].

### PCR amplification and DNA sequencing

For PCR amplification, we targeted the complete Cyt *b* gene using the primers L14724 and H15915 [42]. PCR reactions were performed in total reaction volumes of 20 μl using a PCR buffer (10 mM Tri–HCl, pH 8.3, and 50 mM KCl), 1.5 mM MgCl_2_, 0.2 mM of each dNTP, 2 pmol of each primer, 5 U of Taq DNA polymerase and 1 μl (~30 ng) of the template DNA. The PCR conditions were initial denaturation at 95°C for 10 minutes, followed by 32 cycles of denaturation at 95°C for 45 seconds, annealing at 52–56°C for 45 seconds, and extension at 72°C for 75 seconds. The final extension was at 72°C for 10 minutes. The effectiveness and consistency of the PCR reactions were monitored using positive controls. The amplified PCR amplicons were visualized in UV light on 2% agarose gel stained with ethidium bromide. Exonuclease I (EXO-*I*) and shrimp alkaline phosphatase (SAP) treatments were given to the amplified PCR products (USB, Cleveland, OH) for 15 minutes each at 37°C and 80°C, respectively, to eliminate any residual primer. The amplified PCR products were directly sequenced using the BigDye^®^ Terminator Kit (v3.1) and analyzed on an ABI 3500XL Applied Biosystems Genetic Analyzer. All the products were sequenced in both directions. The sequences were aligned and edited using Sequencer 4.7 (Gene Code Corporation). All the raw sequences were aligned using CLUSTAL W, as implemented in the BioEdit v 7.2.5 software (http://www.mbio.ncsu.edu/BioEdit/bioedit.html).

### Microsatellite genotyping

Eleven microsatellite markers were selected for the analysis: MFW 11, MFW 17 and MFW 26 [18]; BARB 37 and BARB 59 [19]; TPM01, TPM04, TPM15B, TPM11, TPM18B and TPM 21A [20]. Each forward primer was labeled with a fluorescent dye for fragment visualization. The 10 μl multiplex PCR contained 5 μl of Qiagen Multiplex PCR Master Mix (Qiagen Inc., Hilden, Germany) and 0.5 μl of Q solution. Multiplex PCR reactions were carried out in reactions of total volume 10 μl containing 5 μl of Qiagen Multiplex PCR Buffer Mix (Qiagen Inc.), 0.5μl of Q solution, 2 pmol of labeled forward primer, 2 pmol of unlabelled reverse primer (Applied Biosystems), and 20–40 ng of the genomic template DNA. The PCR cycle was performed under the following conditions: initial denaturation at 95°C for 15 minutes, followed by 32 cycles at 95°C for 45 seconds, annealing at 52–58°C for 45 seconds, and extension at 72°C for 90 seconds, with a final extension of 60°C for 30 minutes. The alleles were determined in an ABI 3500XL Genetic Analyzer (Applied Biosystems) using the LIZ 500 Size Standard (Applied Biosystems) and analyzed using GeneMapper version 3.7 (Applied Biosystems).

### Data analysis

#### Mitochondrial DNA

The sequences obtained from the forward and reverse directions were aligned and edited using SEQUENCHER^®^ version 4.9 (Gene Codes Corporation, Ann Arbor, MI, USA). The analysis of each sequence was performed separately using the CLUSTAL X multiple sequence alignment program [43], and the alignments were examined by visual inspection. DnaSP 5.0 [44] was used to analyze the haplotype diversity (h), nucleotide diversity (p), and polymorphic sites (s). The spatial distribution of the haplotypes was visualized through a median-joining network, which was created using the PopART software [45].

To determine whether the GM populations carried a signal of spatial range expansion or a stationary population history, Tajima’s D [46] and Fu’s Fs [47] neutrality test was performed in DnaSP 5.046. To generate the trends in spatial demography history, the mismatch analysis was carried out using the population growth-decline model in DnaSP [44] and to evaluate the fit of the observed distribution the sum of squared deviations (SSD), and the raggedness index (r) under the growth-decline model for each population was used using the ARLEQUIN v3.5 program [48]. The *P*-values were obtained from 1000 simulations on the basis of a selective neutrality test.

#### Microsatellite analysis

The number of alleles per locus (Na), the number of effective alleles (Ne), observed heterozygosity (*Ho*), and expected heterozygosity (*HE*) were estimated using the GenAlEx v6.5 program [49]. Inbreeding (*F*_*IS*_) was calculated in Fstat 2.9.3.2 for each population, and its significance was tested, assuming no Hardy-Weinberg equilibrium within the samples and using 1,000 permutations [50]. The polymorphic information content (PIC) values were calculated using CERVUS v3.0.7 [51]. The supporting format of the data input files was prepared using CONVERT v1.31 [52]. All the loci were checked for under HWE in GenAlEx v6.5 [49]. The pairwise *F*_ST_ values (gene flow) among the populations were calculated using GenAlEx v6.5 [49]. DAPC implement through the *adegenet* package in R was used to determine the genetic structure of the population [53]. DAPC is a multivariate and model-free approach that constitutes an alternative method to validate the individual Bayesian clustering. DAPC provides an efficient description of genetic clusters using a few synthetic variables, called discriminant functions. DAPC seeks linear combinations of the original variables (alleles) which show differences between groups while minimizing variations within clusters. This analysis does not require a population to be in HWE.

The recent population bottleneck signature for microsatellite markers was obtained by testing the deviations of the expected heterozygosity (*HE*) from the heterozygosity expected as the drift-mutation equilibrium (Heq). BOTTLENECK v. 1.2.02 [31] program was run with 1000 iterations under two mutation models: TPM and SMM. The TPM was set with 90% SMM with a variance of 12. Wilcoxon sign-rank test and Mode shift test were used to identify the heterozygosity excess and the allele frequency distributions that discriminate recently bottlenecked from stable populations, respectively [54, 55]. We also calculated the G-W index using ARLEQUIN v3.5 [48], which is a mean ratio of the numbers of observed alleles to all the potential repeats within the allele size range, across all loci, and can detect population bottlenecks from the past [56]. BayesAss v.1.3 program was used to estimate the recent migration rates (past few generations) between the four analysed populations using MCMC (Markov chain Monte Carlo) [57]. We used burn-in iterations 1,000,000 followed by 3,000,000 iterations and a sampling frequency of 2000. The initial run was performed with the default delta (Δ) value 0.15 for allele frequencies (A), migration (M), and inbreeding coefficient (F). Further, the final input parameter of ΔM was adjusted at 0.2. The changes in these parameters would be accepted between 40 and 60%, as recommended by Faubet et al. (2007) [58]. Four independent runs were also performed to validate the consistency of the results.

### Genetic differentiation

The pairwise *F*_ST_ values (gene flow) among the populations were calculated using GenAlEx v6.5 [49]. Pairwise genetic distances between populations were calculated using Nei’s standardized genetic distance *Da*, and the non-rooted tree was generated using the neighbour-joining (NJ) method with 1,000 bootstrap replicates in POPTREE2 [59].

The analysis of molecular variance (AMOVA) was run to estimate the source of the variation found among groups (*F*_CT_), among populations within groups (*F*_SC_), and within populations (*F*_ST_) for the mtDNA and microsatellite markers in ARLEQUIN v3.5 [48]. Estimates of the significance were obtained from 10,000 permutations. A spatial analysis of molecular variance (SAMOVA) was calculated using SAMOVA 2.0 based on sequences data [60]. This approach defines groups of populations that are geographically homogeneous and maximally differentiated from each other. Our analyses were based on 100 simulated annealing steps and a prior definition of the number of groups, K, ranging from 2 to 8. The configuration with the largest associated *F*_CT_ and minimal positive *F*_SC_ values obtained after the 100 independent simulated annealing processes was retained as the best grouping of populations. Additionally, Isolation-by-distance (IBD) was evaluated for the golden mahseer populations using a Mantel test with 1000 permutation as implemented in Alleles In Space version 1.0 based on microsatellite markers [61]. Geographic distances (km) were taken as straight-line distances between localities.

## Supporting information

S1 Fig Bayesian (MCMC) consensus tree of 83 golden mahseer haplotypes based on mtDNA cyt *b* region. Posterior values are provided at their respective nodes. The *Schizothorax richardsonii* (AP011208) was used as outgroup. Asterisk represent the core haplotypes (Hap1 to Hap4).
(DOCX)

S1 Table Comparison of allele size range of nine microsatellite markers with original source used on *Tor putitora*. (DOCX)

S2 Table Sampling locations of golden mahseer in four rivers. N, number of samples.
(DOCX)
