# Supplementary material for: Evaluation of the effect of longitudinal connectivity in population genetic structure of endangered golden mahseer, Tor putitora (Cyprinidae), in Himalayan rivers: Implications for its conservation
Source: PLoS One. 2020 Jun 15;15(6):e0234377. doi: 10.1371/journal.pone.0234377 (PMC7295198; doi:10.1371/journal.pone.0234377)
Supplement: S1 Fig — Posterior values are provided at their respective nodes. The Schizothorax richardsonii (AP011208) was used as outgroup. Asterisk represent the core haplotypes (Hap1 to Hap4). (DOCX) [file pone.0234377.s001.docx]

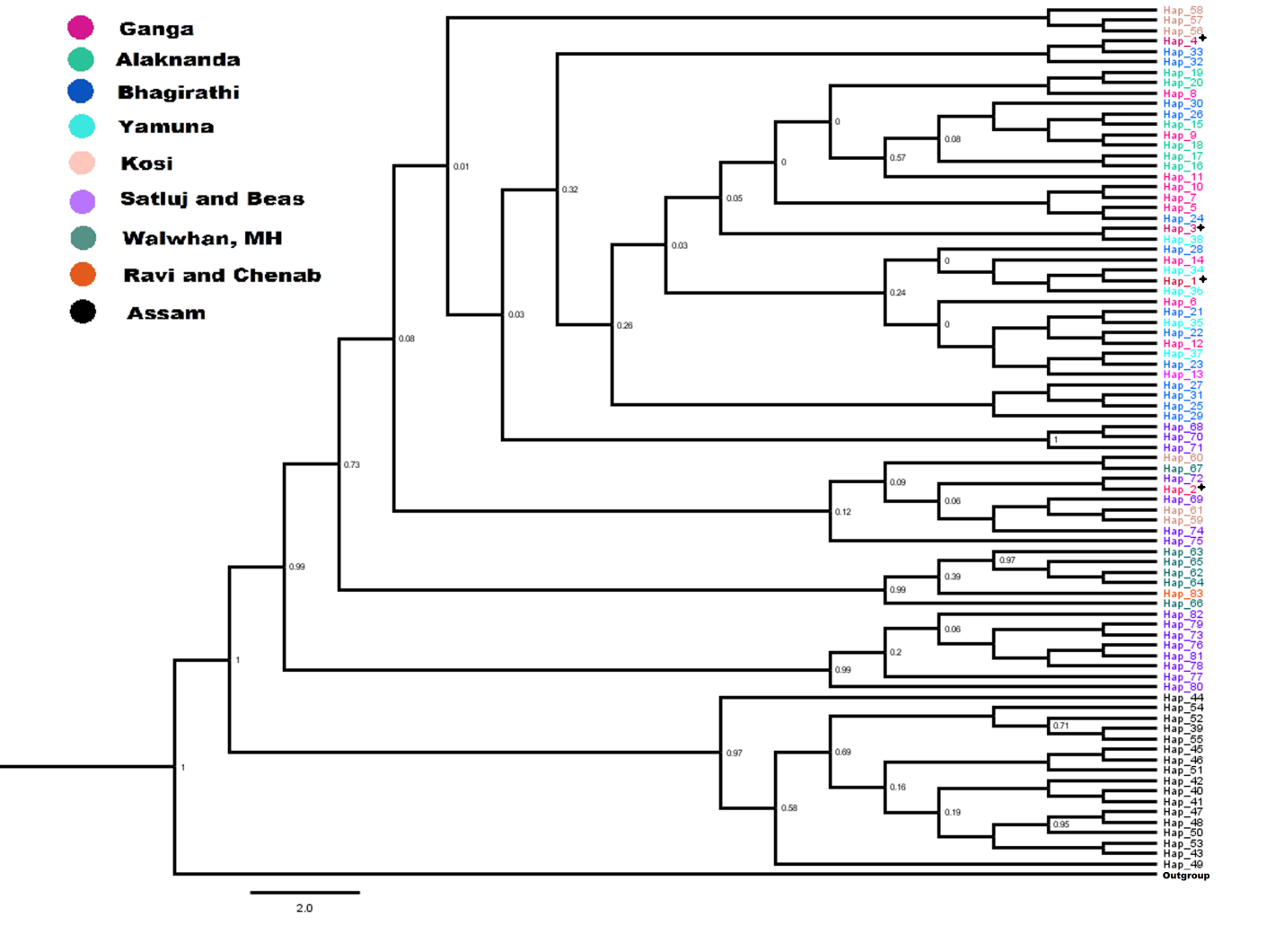


**Supplementary Figure SF 1**. Bayesian (MCMC) consensus tree of 83 golden mahseer haplotypes based on mtDNA cyt *b* region. Posterior values are provided at their respective nodes. The *Schizothorax richardsonii* (AP011208) was used as outgroup. Asterisk represent the core haplotypes (Hap1 to Hap4).
