# Supplementary material for: Evaluation of the effect of longitudinal connectivity in population genetic structure of endangered golden mahseer, Tor putitora (Cyprinidae), in Himalayan rivers: Implications for its conservation
Source: PLoS One. 2020 Jun 15;15(6):e0234377. doi: 10.1371/journal.pone.0234377 (PMC7295198; doi:10.1371/journal.pone.0234377)
Supplement: S1 Table — (DOCX) [file pone.0234377.s002.docx]

**Supplementary Table ST1** Comparison of allele size range of nine microsatellite markers with origin source used on *Tor putitora*.

| **Loci** | **Developed for Species** | **Reported allele size** | **Observed allele size in *Tor putitora*** |
| --- | --- | --- | --- |
| **MFW11**^†^ | *Cyprinus carpio* | 202 | 185-211 |
| **MFW17**^†^ |  | 315 | 210-252 |
| **MFW26**^†^ |  | 136 | 121-175 |
| **BARB37**^*^ | *Barbus* | Not reported | 211-271 |
| **BARB59**^*^ |  |  | 116-156 |
| **TPM01^#^** | *Tor putitora* | 232–278 | 250-288 |
| **TPM11^#^** |  | 136–146 | 120-156 |
| **TPM18B^#^** |  | 150–172 | 150-172 |
| **TPM21A^#^** |  | 122–142 | 102-118 |

^†^ Crooijmans et al., 1997; ^*^ Chenuil et al., 1999, ^#^Sahoo et al., 2013
