# Supplementary material for: Evaluation of the effect of longitudinal connectivity in population genetic structure of endangered golden mahseer, Tor putitora (Cyprinidae), in Himalayan rivers: Implications for its conservation
Source: PLoS One. 2020 Jun 15;15(6):e0234377. doi: 10.1371/journal.pone.0234377 (PMC7295198; doi:10.1371/journal.pone.0234377)
Supplement: S2 Table — N, number of samples. (DOCX) [file pone.0234377.s003.docx]

**Supplementary Table ST2:** Sampling locations of golden mahseer in four rivers. N, number of samples

| **River** | **Sampling sites** | **N** | **Latitude** | **Longitude** |
| --- | --- | --- | --- | --- |
| **Ganga river** | Kinsur, Uttarakhnad (UK) | 1 | 78.5661 | 30.0613 |
|  | Nayar River, UK | 5 | 78.5987 | 30.0634 |
|  | Vyas Ghat, UK | 4 | 78.6038 | 30.0678 |
|  | Nayar-Ganga confluence, UK | 21 | 78.5977 | 30.0607 |
|  | Kangari below, Bhimgoda barrage, UK | 3 | 78.1646 | 29.9054 |
|  | Raiwala, UK | 7 | 78.2338 | 30.2071 |
|  | Bijinor, Uttar Pradesh | 1 | 78.0481 | 29.3919 |
| **Alkananda river** | Srinagar, above dam, UK | 10 | 78.8201 | 30.2378 |
|  | Srinagar, below dam, UK | 16 | 78.8069 | 30.2189 |
| **Bhagirathi river** | Upper Tehri dam, UK | 30 | 78.4409 | 30.3829 |
|  | Koti Village, Tehri, UK | 9 | 78.4653 | 30.4028 |
|  | Dobra Village, UK | 39 | 78.4342 | 30.4478 |
| **Yamuna river** | Hatnikund upstream of barrage, Himachal Pradesh | 55 | 77.5837 | 30.3175 |
